# Central Nervous System-Derived Extracellular Vesicles as Biomarkers in Alzheimer’s Disease

**DOI:** 10.3390/ijms26178272

**Published:** 2025-08-26

**Authors:** Yiru Yu, Zhen Wang, Zhen Chai, Shuyu Ma, Ang Li, Ye Li

**Affiliations:** 1 Key Laboratory of Shaanxi Province for Craniofacial Precision Medicine Research, College of Stomatology, Xi’an Jiaotong University, Xi’an 710049, China; yy9962@stu.xjtu.edu.cn (Y.Y.); wangzhen2024@stu.xjtu.edu.cn (Z.W.); cz1504887184@stu.xjtu.edu.cn (Z.C.); m787118520@stu.xjtu.edu.cn (S.M.); drliang@mail.xjtu.edu.cn (A.L.); 2 Department of Periodontology, College of Stomatology, Xi’an Jiaotong University, Xi’an 710049, China

**Keywords:** Alzheimer’s disease, central nervous system-derived extracellular vesicles, biomarkers, advanced technology

## Abstract

Alzheimer’s disease (AD) has emerged as a global health threat that demands early detection to seize the optimal intervention opportunity. Central nervous system (CNS)-derived extracellular vesicles (EVs), lipid-bilayer nanoparticles released by CNS cells, carry key biomolecules involved in AD pathology, positioning them as a promising source of biomarkers for early detection. Current breakthroughs in EV-based isolation and detection technologies have opened up the possibility of early, accurate AD diagnosis. This review summarizes their multifaceted roles in AD pathogenesis, including amyloid-β (Aβ) aggregation, tau propagation, neuroinflammation, and synaptic dysfunction, and highlights neuron- and glia-derived EV biomarkers with translational potential. We further outline recent advances in EV isolation techniques—including density-, size-, charge/dielectric-, immunoaffinity-, and acoustics-based approaches—and emerging detection platforms such as fluorescence, surface plasmon resonance (SPR), surface-enhanced Raman spectroscopy (SERS), electrochemical, and nanomechanical sensors for sensitive, multiplex AD diagnostics. Finally, we discuss key challenges, including standardization, sensitivity, and high-throughput adaptation, and explore future directions such as automated microfluidics and single-vesicle analysis. CNS-derived EVs hold significant promise as minimally invasive, next-generation tools for early AD detection and precision medicine.

## 1. Introduction

Alzheimer’s disease (AD) is the most prevalent neurodegenerative disease and the principal contributor to dementia worldwide [1]. AD can be conceptualized as a continuum, beginning with an insidious onset, referred to as preclinical AD, gradually progressing to mild cognitive impairment (MCI), and eventually developing into dementia [2]. Notably, degenerative changes in brain tissue—that is, the accumulation of amyloid-β (Aβ) plaques and neurofibrillary tangles (NFTs) formed from phosphorylated tau (p-tau)—precede cognitive symptoms by up to 20 years [3,4]. This chronic progression highlights a need for interventions during the preclinical AD stage, since irreversible damage to nerve cells is limited before the occurrence of clinical symptoms [5]. Delayed diagnosis resulting from traditional methods of detection, such as clinical evaluation and neuropsychological assessment, misses the opportunity for intervention to mitigate disease progression. As such, seizing the optimal intervention opportunity, namely early detection, plays a crucial role in the fight against AD.

The advent of AD biomarkers has created essential detection tools to identify individuals who have Alzheimer’s pathology before symptoms have begun, thereby revolutionizing diagnostic workup of AD [6,7]. An Alzheimer’s biomarker is an objective indicator that accurately reflects the state of disease progression, mainly derived from body fluids [8]. Although dozens of biomarkers have been reported as potential tools for early detection, most still face challenges in their applicability to clinical populations, such as poor stability and limited informational value regarding the disease [9]. It is known that body fluids are not merely repositories of free biomolecules; they also harbor a considerable amount of biological information encapsulated in specialized structures [10]. These structures shield the internal biomolecules from dilution and degradation, maintaining the stability and integrity of information [11]. Central nervous system (CNS)-derived extracellular vesicles (EVs), cell-derived nanosized particles, encapsulate a variety of cargoes (e.g., proteins, lipids, and nucleic acids) in bilayer phospholipid membranes, providing comprehensive and steady molecular information. There is accumulating evidence that CNS-derived EVs propagate neurotoxic proteins and play a crucial role in neuroinflammation, neuronal cell death, and other AD-associated pathological processes [12]. Critically, their lipid bilayer not only shields these labile biomarkers from degradation and dilution but also preserves their cell-type-specific origin, enabling tracking of neuronal injury, astrocytic reactivity, and microglial activation [13]. More importantly, because of their nanoscale size, CNS-derived EVs can cross the blood-brain barrier (BBB), facilitating the protected transport of key cargoes into body fluids, such as plasma, cerebrospinal fluid (CSF), saliva, and urine, among others [14]. Considering these characteristics, CNS-derived EVs hold immense potential as a source of promising biomarkers [15].

Recent years have seen tremendous strides in the field of discovering EV-associated biomarkers [16,17]. Driven by this progress, the technology for the isolation and detection of EV-associated biomarkers has shown significant advancements [18,19]. Conventional isolation methods, such as density-based separation, size-based separation, and precipitation techniques, while widely applied, are often restricted in clinical applications [20]. Traditional detection techniques for EV analysis, including Western blotting, enzyme-linked immunosorbent assay (ELISA), and polymerase chain reaction (PCR), have shortcomings such as low sensitivity and limited ability to detect novel or unknown biomarkers [21]. Over the past decade, extensive efforts have been committed to enhancing EV isolation and detection methods by leveraging acoustic technology, surface plasmon resonance (SPR), surface-enhanced Raman spectroscopy (SERS), electrochemical technology, nanomechanical technology, and other technologies [10].

Herein, we review recent advances and challenges in utilizing CNS-derived EVs as novel biomarkers for AD. First, we outline the molecular mechanisms underlying the involvement of CNS-derived EVs in AD pathogenesis and summarize candidate biomarkers associated with these vesicles. We then provide an overview of technologies for isolating and detecting EV-associated biomarkers, highlighting widely used conventional methods and key emerging methods. Finally, we discuss current challenges in clinical practice and offer a perspective on future directions for their potential clinical application.

## 2. Biological Basis and Clinical Potential of EVs

EVs represent a heterogeneous population of membrane-bound nanoparticles secreted by virtually all cell types [11]. The discovery timeline of EVs traces back to 1946 when Chargaff and West first observed platelet-derived particles during plasma centrifugation for clotting factor isolation [22]. This seminal observation laid the foundation for subsequent EV characterization. The next advancement came in 1967 with Wolf’s electron micrographs of “platelet dust”, revealing the physical characteristics of these vesicles [23]. The 1980s witnessed crucial breakthroughs: Harding et al. demonstrated that intraluminal vesicles are released from multivesicular bodies (MVBs) upon fusion with the plasma membrane during reticulocyte maturation, while Pan et al. subsequently defined these vesicles as “exosomes” and hypothesized their role as a cellular waste disposal mechanism [24,25]. This perspective was fundamentally challenged when emerging studies revealed EVs’ multifaceted biological functions, including enzymatic activity modulation, antigen presentation, functional transfer of mRNAs and microRNAs (miRNAs), and tumor-suppressive effects [26,27]. These paradigm-shifting discoveries not only redefined our understanding of EV biology but also highlighted their diagnostic potential as indicators of physiological and pathological processes, catalyzing exponential growth in EV research [28].

Current EV classification systems integrate biogenesis mechanisms and physical characteristics [11]. Three principal subtypes are recognized: (i) Exosomes (30–150 nm) originate from the endosomal system, in which multivesicular bodies transport intraluminal vesicles to the plasma membrane for extracellular release upon membrane fusion; (ii) Microvesicles (100–1000 nm) form via outward budding and fission of the plasma membrane; (iii) Apoptotic bodies (50–5000 nm) arise from membrane blebbing and fragmentation during apoptosis. To promote standardization, the International Society for Extracellular Vesicles introduced a size-based categorization in its 2018 MISEV guidelines, distinguishing small EVs (sEVs < 200 nm) from large EVs (lEVs > 200 nm) [29].

The diagnostic significance of EVs stems from their molecular cargo—proteins, nucleic acids, lipids, and metabolites—that mirror parental cell physiological states. Clinically, EVs isolated from biofluids (e.g., plasma, serum, saliva, urine, and breast milk) are rich sources of biomarkers, particularly for neurodegenerative diseases [30]. Given these general properties of EVs, the next sections will explore how CNS-derived EVs are involved in AD and how they can be harnessed for diagnosis.

## 3. The Role of CNS-Derived EVs in AD Pathogenesis

AD is a progressive neurodegenerative disorder defined by the accumulation of misfolded proteins and consequent neuronal damage. The two classic neuropathological hallmarks of AD are Aβ peptide plaques and NFTs composed of hyperphosphorylated tau [4]. Aβ, derived from the amyloid precursor protein (APP), aggregates into oligomers and fibrils that deposit as senile plaques in the brain parenchyma [31]. P-tau, a microtubule-associated protein, undergoes abnormal hyperphosphorylation and forms insoluble filaments that aggregate into NFTs within neurons [32]. These processes are accompanied by synaptic loss, neuronal death, and activation of microglia and astrocytes, all of which contribute to cognitive decline. AD pathology typically spreads in a spatiotemporal pattern, suggesting a propagative mechanism by which pathological proteins disseminate through the brain’s networks [33].

**Figure 1:**
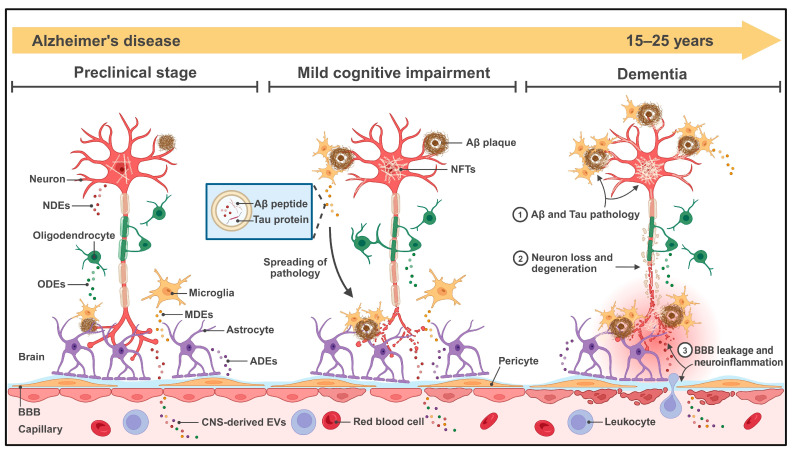
Major CNS cell types (neurons, astrocytes, microglia, oligodendrocytes) secrete EVs which contain Aβ peptides and hyperphosphorylated tau, accelerating the spread of amyloid plaques and NFTs. CNS-derived EVs also transport toxic proteins, regulatory RNAs, inflammatory mediators, and other pathogenic cargo, exacerbating synaptic dysfunction, neuroinflammation, and BBB leakage. Some CNS-derived EVs cross the BBB into peripheral circulation, linking brain pathology to potential biofluid-based biomarkers. Abbreviations: NDEs, neuron-derived extracellular vesicles; ODEs, oligodendrocyte-derived extracellular vesicles; MDEs, microglia-derived extracellular vesicles; ADEs, astrocyte-derived extracellular vesicles; BBB, blood-brain barrier; NFTs, neurofibrillary tangles. (Image created with BioRender.com, with permission).

Mounting evidence indicates that EVs play an increasingly important role in the propagation of Aβ and tau pathology [34]. In the CNS, neurons and glial cells continuously release EVs under physiological and pathological conditions. These EVs can travel through interstitial fluid and across synaptic connections to deliver their cargo to target cells. In neurodegenerative diseases like AD, which are characterized by misfolded protein aggregates, EVs have emerged as key players in the prion-like spread of pathology. Pathogenic proteins packaged within EVs are shielded from proteolytic degradation, allowing them to persist and diffuse to distant brain regions. Below, we review how CNS-derived EVs contribute to the biological progression of AD, with a focus on their roles in Aβ and tau pathology, as well as other mechanisms. Figure 1 shows the role of CNS-derived EVs in AD pathogenesis.

### 3.1. CNS-Derived EVs and Aβ Pathology

Aβ peptides are produced through sequential cleavage of APP by β-secretase (BACE1) and γ-secretases [35]. These peptides can aggregate and form amyloid plaques, leading to synaptic dysfunction and neuronal loss. EVs play multifaceted roles in the generation, propagation, and clearance of Aβ pathology. One prominent role of EVs is in Aβ aggregation and plaque formation. CNS-derived EVs are enriched in certain lipids (e.g., GM1 ganglioside) that can act as seeds for extracellular amyloid fibril formation [36]. Studies have reported that EVs accelerate Aβ40 and Aβ42 fibril formation [37]. Similarly, cellular prion protein, which is highly enriched on EVs from neuroblastoma cells, can bind Aβ oligomers and promote their polymerization into less soluble fibrils. The binding of EVs to Aβ oligomers may reduce the acute neurotoxicity of soluble Aβ species at synapses, but it concurrently facilitates the growth of insoluble plaques [38]. These observations support that EVs can actively promote Aβ aggregation and plaque formation.

Beyond seeding aggregates, EVs contribute to Aβ propagation. EVs can carry key molecules involved in Aβ production: APP, BACE1, γ-secretase subunits, as well as cleavage products, including soluble APP fragments and Aβ peptides, have all been identified in CNS-derived EVs [34,39]. In addition to Aβ production via APP cleavage, the transport of Aβ peptides by EVs constitutes a key mechanism for pathological spread. In the AD brain, accumulated Aβ is loaded into EVs, which then propagate these toxic peptides in a prion-like mechanism. Notably, sEVs from AD patients’ brains carry elevated amounts of Aβ oligomers and facilitate the transfer of this toxic cargo between neurons [40]. EVs can impair neuronal calcium homeostasis and mitochondrial function, sensitizing neurons to excitotoxicity [41]. In vivo studies reported that pharmacological inhibition of EV biogenesis using neutral sphingomyelinase inhibitors such as GW4869 reduced exosome production and was associated with lower amyloid plaque load in the 5XFAD mouse model of AD [42]. Furthermore, EVs are implicated in Aβ clearance mechanisms: they can serve as vehicles that remove Aβ from the extracellular space or facilitate its degradation. Neuron-derived extracellular vesicles (NDEs) bearing Aβ are efficiently taken up by microglia, which then degrade the Aβ cargo within lysosomes. Moreover, microglial exosomes expressing the receptor TREM2 facilitate Aβ clearance by binding Aβ and promoting its phagocytosis through modulation of the local inflammatory environment [43]. In addition, microglia secrete EVs that carry enzymes and factors important for Aβ catabolism. For example, microglia-derived extracellular vesicles (MDEs) contain insulin-degrading enzyme (IDE) and neprilysin, which are proteases known to break down Aβ peptides [44]. By releasing EVs enriched in Aβ-degrading enzymes into the brain interstitium, microglia can help digest extracellular Aβ aggregates. In summary, CNS-derived EVs play multiple roles in Aβ pathology—fostering amyloid spread and aggregation in some circumstances, but aiding Aβ removal in others. The net impact of EVs likely depends on their cellular origin and cargo composition, which are influenced by the stage of disease and the brain’s inflammatory milieu.

### 3.2. CNS-Derived EVs and Tau Pathology

Tau pathology in AD exhibits a stereotyped spreading pattern, beginning in the entorhinal cortex (Braak stages I–II), subsequently involving limbic areas (Braak stages III–IV), and ultimately reaching neocortical regions (Braak stages V–VI) [33]. This hierarchical pattern of spread suggests the trans-synaptic propagation of pathogenic tau species along neuronal circuits. In a cryo-electron tomography study of EVs from AD brain tissue, truncated tau filaments—including paired helical filaments and straight filaments—were identified as tethered structures within EV lumens [45]. These tau-laden EVs can disperse in the brain interstitial fluid or CSF and be internalized by neighboring cells. A growing body of research implicates CNS-derived EVs as facilitators of tau propagation. Neurons containing hyperphosphorylated tau can secrete tau-bearing EVs that are taken up by connected neurons, thereby transmitting tau pathology from one region to the next. Importantly, EV encapsulation appears to protect tau from degradation and immune clearance, preserving its seeding activity during transit. This mechanism enables minute amounts of pathogenic tau to initiate aggregation cascades in recipient cells, akin to prion-like seeding.

Multiple lines of evidence confirm that EVs actively drive tau spread. Tau is internalized by cellular clearance systems but, rather than being fully degraded, is subsequently encapsulated into EVs [46]. Significantly, these EVs facilitate the systemic propagation of tau pathology across neural networks. Microglia are capable of taking up and releasing tau, and inhibition of EV synthesis or microglial depletion significantly attenuates tau pathology propagation in an AD mouse model [47]. Supporting this mechanism, oral administration of the P2RX7 receptor inhibitor GSK1482160 suppresses MDE secretion, reduces hippocampal accumulation of misfolded tau, and improves cognitive deficits in a tauopathy AD mouse model [48]. These findings highlight EVs as necessary mediators of abnormally p-tau spreading. Notably, CNS-derived EVs from AD patients have been shown to be highly potent in inducing tau spreading in vivo: when injected into the mouse brain, tau-loaded EVs triggered widespread tau pathology in recipient neurons, predominantly in hippocampal GABAergic interneurons, whereas equivalent amounts of free tau oligomers or fibrils caused minimal effect [49]. This suggests that the EV context not only preserves tau seeds but may also target them to specific cell types. The precise targeting mechanism is still under investigation, but EV surface molecules likely influence which cells and subcellular compartments take up the tau cargo.

### 3.3. CNS-Derived EVs and Other Mechanisms in AD Progression

Beyond Aβ and tau pathology, CNS-derived EVs influence several other pathogenic mechanisms in AD. Chronic neuroinflammation is a prominent feature of AD, and EVs are important mediators of neuron-glia communication in this context. EVs can propagate inflammatory signals by carrying cytokines, chemokines, and danger-associated molecules between cells [50]. Moreover, EVs carry miRNAs that regulate inflammatory pathways. Evidence indicates that PC12-derived exosomes carrying miR-21-5p are phagocytosed by microglia, triggering M1 polarization [51]. Synapse loss and dysfunction are early events in AD that correlate strongly with cognitive decline. EVs contribute to synaptic pathology by transferring toxic proteins and regulatory RNAs that alter synaptic structure and function [52]. Inflammatory microglia release EVs containing miRNAs (e.g., miR-146a-5p) which control the expression of presynaptic synaptotagmin-1 and postsynaptic neuroligin-1. This leads to dendritic spine loss accompanied by a decrease in the density and strength of excitatory synapses [53]. A growing area of AD research is the role of BBB dysfunction in disease progression. The BBB normally shields the brain from peripheral toxins and regulates immune cell entry; in AD, the BBB becomes leaky and less regulated. Recent evidence suggests EVs can influence BBB integrity. Specifically, M1-polarized microglia-derived exosomes exacerbate BBB dysfunction by delivering neuroinflammatory miRNAs that suppress tight junction proteins and increase endothelial permeability [54]. Overall, EVs influence the pathogenesis of Alzheimer’s disease via multiple mechanisms. Understanding these mechanisms underscores the importance of developing reliable CNS-derived EV biomarkers and necessitates robust isolation and detection methods, discussed in the following sections.

## 4. Promising CNS-Derived EV Biomarkers in AD Progression

AD progression encompasses a spectrum of neuropathologic changes, ranging from Aβ aggregation and tau propagation to synaptic dysfunction and glial activation [55]. CNS-derived EVs have been found to contain pathological proteins, inflammatory molecules, transcription factors, and nucleic acids that correlate with AD stage and cognitive decline. Below, we summarize key EV biomarkers originating from CNS cell types such as neurons, astrocytes, microglia, and oligodendrocytes, and delineate their roles across AD pathology. Table 1 provides a summary of published studies on potential CNS-derived EV biomarkers in the detection of AD.

### 4.1. NDE Biomarkers

Neurons are the principal functional units of the CNS, responsible for processing, transmitting, and integrating information via synaptic connections [83]. They play an essential role in cognitive functions such as learning and memory. In AD, neuronal dysfunction and death are the primary pathological substrates underlying cognitive decline [2]. Given the central role of neurons in AD pathogenesis, NDEs circulating in peripheral blood offer an opportunity to monitor molecular changes occurring within neurons during disease progression. A growing body of literature indicates that NDE cargo changes in accordance with AD pathophysiology [12].

NDEs have demonstrated a variety of AD-related biomarkers, reflecting core hallmarks like Aβ and tau pathology. Winston et al. showed that plasma NDEs from patients with AD or MCI who later converted to AD contain elevated levels of P-T181-tau, P-S396-tau, and Aβ1-42, compared to cognitively normal controls and stable MCI patients. Notably, these abnormal NDE protein profiles predicted conversion from MCI to AD dementia accurately. In fact, NDEs from AD patients could even seed tau pathology when injected into normal mice, underscoring the pathogenic potential of their cargo [56]. Jia et al. similarly demonstrated elevated Aβ42, T-tau, and P-T181-tau in NDEs from AD/MCI patients versus controls, with these levels strongly correlating with CSF biomarker levels [57]. More recent work by Li et al., in a large Chinese cohort, showed NDE Aβ42 rises progressively from cognitively normal amyloid-negative individuals to amyloid-positive individuals, through MCI, and into AD dementia. Furthermore, NDE Aβ42 correlated with positron emission tomography (PET) imaging measures of amyloid burden and predicted longitudinal cognitive decline and entorhinal cortex atrophy [58]. Importantly, multiple studies confirm that NDE levels of p-tau and Aβ can predict future AD diagnosis with high accuracy, up to 5–10 years before symptom onset. For instance, Kapogiannis et al. reported that a composite of NDE biomarkers—Aβ42, p-tau, and phosphorylated insulin receptor substrate 1 (IRS-1)—could predict the development of AD in cognitively normal individuals nearly a decade in advance [63]. These observations establish NDE-derived tau and Aβ as promising early biomarkers of AD progression.

Synaptic dysfunction and loss are early features of AD that are not directly captured by classic Aβ/tau biomarkers. NDEs provide a unique window into synaptic health. A consistent finding across studies is that levels of certain synaptic proteins in NDEs decline as AD progresses, mirroring synaptic damage. Goetzl et al. reported significantly lower NDE levels of synaptophysin, synaptopodin, synaptotagmin-2, neurogranin (NRGN), and growth-associated protein 43 (GAP43) in AD patients compared to controls. Crucially, NDE synaptic protein levels correlated strongly with cognitive scores, whereas NDE Aβ42 and P-T181-tau did not correlate with cognitive scores [65]. This suggests NDE synaptic markers may be particularly sensitive indicators of functional neuronal integrity and disease severity. Follow-up studies reinforced these findings. Jia et al. found that concentrations of GAP43, NRGN, synaptosome-associated protein 25 (SNAP25), and synaptotagmin-1 in NDEs were significantly lower in AD patients compared to controls, and that their decline in NDEs reflects the synaptic loss that accompanies early AD. A combination of these synaptic markers could predict progression to AD 5–7 years before cognitive impairment [66]. Consistently, Winston et al. noted that NRGN was significantly lower in AD and in MCI patients who converted to AD, compared to controls [56]. Moreover, Goetzl et al. found that NDE levels of several functionally specialized synaptic proteins—neuronal pentraxin 2 (NPTX2), neurexin 2α (NRXN2α), AMPA4-containing glutamate receptor (AMPA4), and neuroligin 1 (NLGN1)—decline progressively with AD advancement and cognitive loss [67]. Taken together, the loss of synaptic protein cargo in NDEs reflects synapse degeneration, beginning in preclinical stages and worsening with disease progression.

Among the novel AD biomarkers identified in CNS-derived EVs, proteomic studies have uncovered several noteworthy candidates. In a quantitative mass-spectrometry analysis, Zhong et al. proposed complement component 7 (C7) and zyxin (ZYX) as EV biomarkers that showed expression changes from normal to MCI to AD in NDEs, suggesting they could serve as potential preclinical indicators of impending cognitive decline [72]. Arioz et al. further identified a significant enrichment of hemoglobin subunits α, β, and δ in NDEs from AD patients compared to healthy controls using LC-MS/MS proteomics. The biological significance of this finding remains unclear; it may reflect BBB disruption or microhemorrhages in AD. Nevertheless, the presence of hemoglobin subunits within NDEs represents a potential novel biomarker [73]. Beyond these proteomics-identified candidates, other emerging NDE-associated biomarkers of AD have been reported through alternative approaches. For instance, NDEs from AD patients show significantly reduced levels and catalytic activities of mitochondrial electron transport chain complexes and of superoxide dismutase 1 (SOD1), highlighting that mitochondrial dysfunction in AD can be detected via EVs [74]. Another emerging biomarker is the repressor element-1 silencing transcription factor (REST), a neuroprotective factor induced in aging that is lost in AD. Winston et al. observed significantly lower REST levels in plasma NDEs of AD and MCI-to-AD converters compared to controls [56]. Alvarez et al. likewise found that NDEs REST levels exhibit stage-dependent changes across AD progression [64].

In addition to proteins, miRNAs in NDEs demonstrate diagnostic value. Durur et al. identified miR-let-7e-5p in NDEs as significantly upregulated in AD, with high diagnostic value. Notably, NDEs enriched in miR-let-7e-5p induced increased IL-6 expression in co-cultured microglia, suggesting that NDE miRNAs can drive neuroinflammatory responses [78]. Another study by Pounders et al. used RNA sequencing of NDEs and found a disease-specific miRNA signature: miR-122 and miR-3591 were significantly downregulated in AD samples compared to healthy controls and frontotemporal dementia, supporting the utility of NDE miRNA profiles for differential diagnosis of neurodegenerative dementias [79].

### 4.2. Glia-Derived EV Biomarkers

Glial cells (astrocytes, microglia, and oligodendrocytes) have emerged as central players in AD pathogenesis, with their activation and dysfunction tightly linked to neuroinflammation, protein clearance, and synaptic support [84]. Notably, EVs derived from these glial cells carry biomarkers that reflect their pathological states, offering novel biomarker potential for tracking AD progression. Astrocytes are critical for maintaining neuronal homeostasis, and their reactivity in AD contributes to inflammation and Aβ metabolism [85]. Astrocyte-derived extracellular vesicles (ADEs) carry distinctive inflammatory and complement proteins that mirror astrocytic activation. Goetzl et al. demonstrated that ADEs from AD patients contain strikingly high levels of complement cascade components—including C1q, C4b, C3b, C3d, fragment Bb, factor B, factor D, and the C5b-C9 terminal complement complex (TCC)—compared to controls. Meanwhile, pro-inflammatory cytokines interleukin (IL)-6, tumor necrosis factor-α (TNF-α), and IL-1β were also elevated in ADEs from AD patients. Intriguingly, follow-up of patients over time showed that complement levels in ADEs increased as AD progressed. In parallel, levels of complement regulatory proteins CD59, CD46, decay-accelerating factor (DAF), and complement receptor type 1 (CR1) were diminished in ADEs from AD patients, indicating a dysregulated complement system in astrocyte EV cargo [69]. This complement signature in ADEs correlates with disease advancement and synaptic damage. In another study, Goetzl et al. showed that ADEs in plasma carry elevated levels of BACE1 and soluble amyloid precursor protein-β (sAPPβ), along with reduced levels of glial cell line-derived neurotrophic factor (GDNF) in AD patients, highlighting the potential of astrocytic EV cargo as accessible biomarkers for detecting and monitoring AD [77]. Furthermore, a study found that monocyte chemoattractant protein-1 (MCP-1) is elevated specifically in ADEs during the subjective cognitive decline (SCD) stage of AD, despite unchanged plasma levels [70]. This suggests that ADE biomarkers may mark the transition to an inflammatory, neurotoxic environment at early, preclinical disease stages.

Microglia are the resident immune cells of the CNS, and their activation states profoundly influence AD progression [86]. MDEs carry biomarkers of microglial status and activity. Kumar et al. identified a distinct miRNA signature in plasma MDEs from AD patients: miR-29a-5p, miR-125b-5p, miR-132-5p, miR-210-3p, and miR-106b-5p within MDEs exhibit distinct, stage-dependent expression alterations in cognitive impairment and demonstrate high diagnostic accuracy for AD progression, correlating with brain structural changes [80]. This distinctive CNS-derived EV miRNA signature underscores these miRNA changes correlated with disease stage and brain atrophy measures, highlighting their promise as accessible biomarkers of neuroinflammation. Meanwhile, proteomic and lipidomic analyses of microglial EVs have identified state-specific molecular changes in AD. Cohn et al. performed a multi-omics analysis of CD11b-positive microglial EVs isolated from postmortem AD brain tissue. They observed a significant reduction in the homeostatic microglial markers P2RY12 and transmembrane protein 119 (TMEM119), along with elevated levels of disease-associated markers ferritin heavy chain (FTH1) and TREM2 in AD patients’ MDEs. MDEs also contained higher tau levels and exhibited a pro-inflammatory lipid profile with elevated free cholesterol and reduced docosahexaenoic acid (DHA)-containing lipids. Additionally, several miRNAs associated with immune activation and cellular senescence pathways were significantly upregulated in AD patients’ MDEs [87]. Collectively, these findings highlight both ADE- and MDE-associated molecular signatures that reflect AD pathology and further support the strong potential of glial cell-derived EVs as diagnostic biomarkers and mechanistic indicators of neuroinflammation in AD.

It is worth noting that oligodendrocyte-derived extracellular vesicles (ODEs) have been less studied in AD, but they may carry myelin-related proteins whose changes could signal white-matter pathology in AD [88]. In summary, CNS-derived EVs from multiple cell types carry a wealth of biomarkers relevant to AD.

To translate these EV biomarkers into clinical practice, robust methods are required to efficiently isolate CNS-derived EVs from complex biofluids and reliably detect their cargo. In the next sections, we review the major technologies for EV isolation and for sensitive detection of EV-associated biomarkers.

## 5. Isolation and Enrichment Technologies for CNS-Derived EVs in AD Diagnostics

Isolating CNS-derived EVs presents unique challenges due to anatomical brain barriers and the low abundance of CNS-origin vesicles in peripheral biofluids. Ensuring high purity and yield is critical, as contaminants or vesicle damage during isolation can confound downstream biomarker analysis. Below, we will categorize and evaluate diverse EV separation techniques used in AD research, focusing on their clinical applicability for detecting AD-specific signals in patient-derived biofluids. Table 2 provides a comparison of CNS-derived EV isolation methods.

### 5.1. Density-Based Separation

Density-based methods isolate EVs using ultracentrifugation, exploiting their buoyant density. In differential ultracentrifugation (dUC), sequential high-speed spins pellet particles by size/density [89]. An alternative is density gradient ultracentrifugation, where EVs float or sediment into a medium (e.g., sucrose or iodixanol) at the position corresponding to their buoyant density [90]. This yields finer separation of EVs from proteins and other particles. Classical dUC has been widely used to isolate EVs from CNS-derived samples [108]. For instance, Chatterjee et al. isolated EVs from human CSF via ultracentrifugation to identify AD-related EV cargo changes. Their results demonstrated that even with very small sample volumes, density-based isolation can yield sufficient EVs for discovering candidate biomarkers [109].

Density-based ultracentrifugation is considered a gold standard for EV purification due to its high-purity output [110]. Density gradient ultracentrifugation removes many of the aggregating vesicles due to the high g-forces. In addition, throughput is low because only a limited number of patient samples can be processed per run. In summary, while density-based methods are invaluable in research and have revealed AD-specific EV cargo, their labor-intensive nature and equipment needs currently limit routine clinical use. Ongoing efforts to miniaturize ultracentrifugation technology or to develop faster alternatives are needed to translate this approach to clinical settings.

### 5.2. Size-Based Separation

Size-based methods separate EVs by their physical size using filtration or chromatographic approaches. One common technique is size-exclusion chromatography (SEC), in which a column packed with porous resin traps smaller molecules and allows larger particles like EVs to elute first. SEC thus fractionates EVs away from the bulk of soluble proteins, yielding relatively intact vesicles [93]. SEC has gained popularity for biofluid EV isolation due to its simplicity and gentleness. For example, Kangas et al. noted that combining ultrafiltration with SEC (UF-SEC) could outperform traditional ultracentrifugation and precipitation in both yield and purity of EVs. Even the smallest volume tested (0.5 mL) yielded ~700 EV-associated proteins after UF-SEC, demonstrating the method’s efficiency for low-volume samples [111]. Another approach is membrane ultrafiltration, using membranes with defined pore sizes to retain larger vesicles while allowing smaller contaminants to pass [94]. Sequential filtration can concentrate an EV-rich fraction. Newer variations include tangential flow filtration, which continuously flushes the sample across the filter to reduce clogging, and microfluidic sieving structures. These size-based methods operate without extreme forces, better preserving EV integrity.

Size-based methods require no elaborate reagents or expensive equipment, making them cost-effective and relatively quick [95]. However, a major limitation is that size-based approaches cannot fully distinguish CNS-derived EVs from other similarly sized particles. For example, lipoproteins in blood often co-elute with sEVs in SEC fractions. In summary, size-based techniques are fast and convenient, but additional steps may be needed to achieve the purity required for reliable biomarker assays [112].

### 5.3. Precipitation Methods

Precipitation methods isolate EVs by reducing their solubility, causing them to sediment out of solution at low centrifugal forces. The most common approach uses hydrophilic polymers like polyethylene glycol (PEG) [96]. PEG, when added to a biofluid, binds water molecules and creates a hydrophobic environment that aggregates EVs, which can then be pelleted by low-speed centrifugation. Polymer-based precipitation is widely used in EV studies because it is simple and requires no special equipment. For instance, Lee et al. examined neuron- and astrocyte-derived EV Aβ and tau. They used a commercial PEG solution (ExoQuick) to precipitate total EVs, followed by immunochemical enrichment of specific EV subpopulations [113]. Another precipitation strategy is protein organic solvent precipitation (PROSPR), which employs organic solvents to precipitate abundant soluble proteins, leaving EVs in the supernatant for collection [114]. By removing competing soluble proteins, EVs become relatively enriched and can be subsequently pelleted or filtered.

Precipitation methods are convenient, inexpensive, and scalable, making them attractive for initial CNS-derived EV enrichment [97]. In a clinical context, precipitation methods could allow high-throughput processing of many samples in parallel. However, the major drawback is co-isolation of contaminants [98]. This can reduce the specificity of biomarker assays and add background noise. Thus, precipitation is best used for initial concentration of CNS-derived EVs, but not as a stand-alone method when high analytical specificity is required.

### 5.4. Charge-/Dielectric-Based Separation

Charge- and dielectric-based techniques leverage the electrical properties of EVs to isolate them [99]. EV membranes carry an inherent negative surface charge due to phospholipids and surface proteins [115]. Electrophoresis can therefore migrate EVs in an electric field toward a positive electrode, separating them from less-charged contaminants. Traditional electrophoresis in gels is not practical for collecting EVs, but free-flow electrophoresis or capillary electrophoresis systems have been explored to separate vesicles by charge. More sophisticated is dielectrophoresis (DEP)—the movement of polarizable particles in a non-uniform electric field. By tuning the electric field frequency, DEP can selectively trap EVs (which have specific dielectric properties) on microelectrodes, separating them from cells or larger debris. DEP chips have been used to isolate exosomes by inducing them to cling to electrode surfaces, achieving enrichment from biofluid [116]. Another method uses electrostatic interactions—cation-exchange resins or charged nanofilters that bind negatively charged EVs. For example, chitosan coated on a scaffold will attract negatively charged EV membranes in an acidic buffer, immobilizing the vesicles by electrostatic adsorption. After binding EVs, the scaffold can be washed to remove unbound impurities [117].

Charge-/dielectric-based methods are gentle and label-free, avoiding harsh forces or antibody reagents and preserving their integrity and cargo functionality [100]. They can be integrated into portable devices and handle small volumes, aligning well with point-of-care needs. However, they often require specialized equipment and are sensitive to the ionic conditions of the buffer. Additionally, like size methods, they might not perfectly discriminate all EV subtypes. Ongoing developments in microfluidic electrophoresis and DEP are showing promise for small-volume samples [101].

### 5.5. Immunoaffinity-Based Separation

Immunoaffinity-based methods isolate EVs by targeting specific surface molecules on the vesicle membrane [118]. Typically, antibodies against EV surface proteins are immobilized on a solid phase. When the sample is applied, EVs displaying the target epitope will bind to the solid phase and can be subsequently eluted or analyzed in situ [119]. The major advantage of immunoaffinity-based methods is their high specificity in enriching EV subpopulations from specific cellular origins or subtypes [102]. NDEs are commonly enriched using L1 cell adhesion molecule (L1CAM, CD171), neural cell adhesion molecule (NCAM), and synaptosomal-associated protein-25 (SNAP25) [68,120,121,122]. For ADEs, glutamate-aspartate transporter (GLAST, EAAT1, ACSA-1), aquaporin-4 (AQP4), and glial fibrillary acidic protein (GFAP) are frequently utilized [123,124,125,126]. ODEs can be captured using myelin oligodendrocyte glycoprotein (MOG), while MDEs are typically isolated using CD11b and TMEM119 [127,128]. Notably, no single marker is perfectly specific: for instance, L1CAM and GLAST are present on multiple cell types. To enhance specificity, researchers are now considering combined markers or novel candidates identified by proteomics [129]. Beyond antibodies, immunoaffinity ligands like aptamers or lectins have been employed. For instance, aptamers can be selected to bind EV surface markers with high affinity and specificity [130]. They offer the advantages of chemical stability and easy modification. Overall, immunoaffinity-based separation is a powerful technique for enriching highly specific EV subsets, and ongoing improvements in surface markers and binding ligands continue to refine its selectivity.

Immunoaffinity-based separation is widely regarded as one of the most effective approaches for achieving high-purity isolation of EVs, as it yields a highly specific subset of vesicles [103]. By targeting markers unique to defined CNS-EV populations, this method enables the enrichment of fractions that are particularly relevant to disease processes. However, immunoaffinity-based separation is often associated with lower yields and requires substantial amounts of antibody-coated beads per sample, contributing to increased costs [104]. Overall, this technique provides targeted enrichment and exceptional purity but at the expense of recovery and cost-efficiency, making it most suitable for clinical biomarker discovery and focused mechanistic studies. With continued optimization, immunoaffinity-based EV isolation holds strong potential for translation into diagnostic assays for AD and other neurodegenerative diseases.

### 5.6. Acoustic Separation

Acoustic separation uses ultrasonic sound waves to manipulate EVs in fluid [105]. When a sample is subjected to a standing acoustic wave (e.g., a microfluidic channel), particles experience an acoustic radiation force that pushes them toward pressure nodes or antinodes depending on their size and density [106]. EVs can be trapped at these nodes in a flowing system. Acoustic trapping devices create a stable ultrasonic field that captures EVs from the fluid while allowing smaller molecules to wash away [131]. By adjusting power and flow, one can enrich EVs and even concentrate them into a small volume. Sattarov et al. reported the use of acoustic trapping to isolate CSF-EVs in AD. Using the AcouTrap2 device, they sonicated 75 µL of CSF in a capillary chamber, trapping EVs on 10 µm silica seed particles within an ultrasound node [132]. Li et al. introduced oscillating microbubble array-based metamaterials (OMAMs), which leverage acoustically excited microbubble oscillation to rapidly isolate high-purity exosomes and size-specific subpopulations from undiluted whole blood in 3 min without labeling or preprocessing [133].

Acoustic separation is gentle, label-free, and efficient for small volumes [107]. It does not alter EVs through chemicals or surface binding. The EVs remain in suspension and can be retrieved intact, which is critical for functional studies. The ability to remove free proteins via washing in the trap means high purity can be achieved without multiple centrifugation or column purification steps. For clinical use, the appeal is that only a small fluid sample is needed, which is especially valuable when the sample is scarce [106]. One challenge lies in ensuring consistent performance for clinical reproducibility, which requires procedures such as the calibration of acoustic nodes. Furthermore, future implementation will require scale-up and automation as the field advances. In conclusion, acoustic separation provides a rapid, non-destructive EV isolation method, enabling the sensitive and specific detection of CNS-derived EV biomarkers in biofluids.

Each isolation method presents trade-offs between yield, purity, scalability, and specificity. In many cases, integrating multiple approaches may offer the best path forward for clinical translation. Addressing current challenges in isolation will directly impact our ability to achieve sensitive and specific detection of CNS-derived EV biomarkers in biofluids.

## 6. New Detection Technologies for CNS-Derived EVs in AD Diagnostics

**Figure 2:**
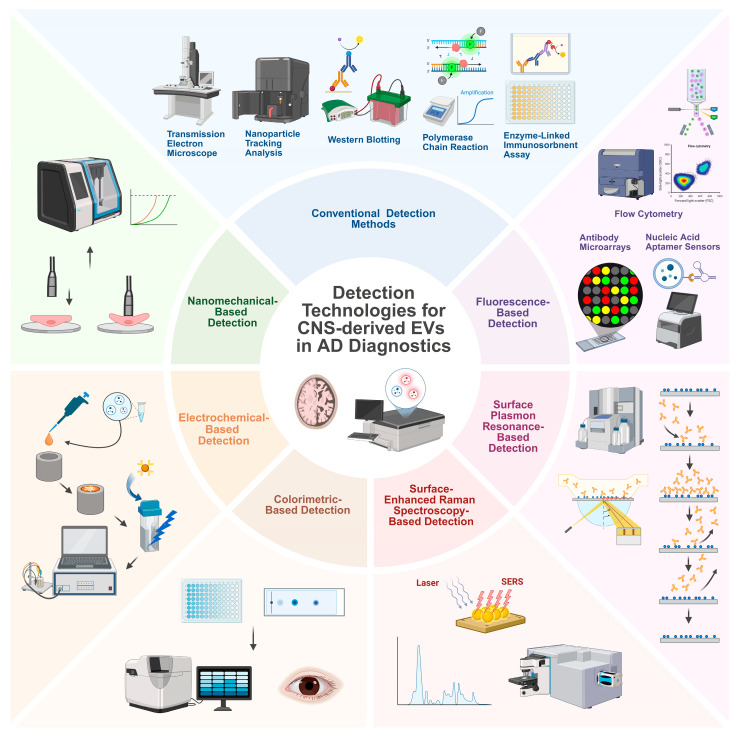
Detection technologies for CNS-derived EVs in AD diagnostics. Detecting and analyzing CNS-derived EVs as biomarkers in AD involves a range of traditional and novel technologies. Major emerging technologies include fluorescence-based detection, surface plasmon resonance-based detection, surface-enhanced Raman spectroscopy-based detection, electrochemical-based detection, colorimetric-based detection, and nanomechanical-based detection. These diverse technologies offer complementary advantages and are being developed to detect CNS-derived EV biomarkers in patient biofluids for early, minimally invasive AD diagnosis. (Image created with BioRender.com, with permission).

Conventional EV detection methods such as Western blotting, PCR, and ELISA remain widely used in clinical research, but they exhibit fundamental limitations that impede their utility in clinical applications [18,19]. Emerging approaches based on optical, electrical, and mechanical sensing platforms have been developed to enable the sensitive and specific detection of CNS-derived EV biomarkers in biofluids [134]. Here, we review several major classes of new detection technologies, highlighting their principles and applications to CNS-EV biomarker detection in AD. Figure 2 provides a summary of detection technologies for CNS-derived EVs in AD diagnostics.

### 6.1. Fluorescence-Based Detection

Fluorescence-based detection methods utilize light-emitting probes to identify and quantify EVs or their cargo with high sensitivity and multiplexing capability [135]. These methods are highly versatile, encompassing various technologies such as flow cytometry (FCM), antibody microarrays, and nucleic acid-based sensors.

#### 6.1.1. FCM

FCM is a laser-based technology enabling high-throughput, multiparameter analysis of individual particles in suspension based on their light scattering properties and fluorescence emission from labeled markers [136]. Applied to EVs, enhanced FCM allows the characterization of individual EVs based on size, complexity, and surface biomarkers. Tian et al. applied high-sensitivity FCM to plasma EVs carrying synaptic function- and brain-related proteins [137]. Similarly, Dayarathna et al. demonstrated the power of multiplexed nano-FCM to capture disease signatures. Using a customized nanoscale flow assay, they screened plasma from individuals with MCI, AD, and controls for EVs bearing multiple antigens [138]. Importantly, this nano-flow assay required only minimal sample volumes and no ultracentrifugation or purification, as EVs were directly labeled in diluted plasma. This finding underscores that FCM can sensitively detect EV biomarkers reflecting core AD pathologies at early and late stages. Additionally, multiplexed bead-based FCM provides an alternative strategy to profile EVs when direct scatter detection is challenging. Brahmer et al. evaluated a commercial MACSPlex EV kit Neuro, in which EVs are first captured onto beads via surface antibodies and then labeled with a panel of fluorescent antibodies. This multiplex flow platform covers 37 different surface markers relevant to CNS-derived EVs and yields a semi-quantitative fingerprint of CNS-derived EVs in the sample [139]. While the sensitivity for any single CNS-specific antigen was limited due to its low abundance and some masking by abundant EV proteins, the multiparametric pattern of EV markers was able to differentiate disease states, highlighting the utility of high-dimensional flow cytometric analysis in neurology.

Enhanced FCM-based methods offer high sensitivity for EV detection. By fluorescently tagging EV surface markers, such methods achieve excellent specificity for cells of origin [140]. Another advantage is throughput: FCM can analyze thousands of particles per second. However, fluorescent labeling improves confidence by only counting events positive for specific markers, but non-specific antibody binding or antibody aggregates can produce false positives if not properly gated [141]. Nevertheless, the ability to directly measure CNS-derived EVs and obtain multi-marker readouts in a single test is a significant advancement. As protocols mature and instruments become more EV-sensitive, enhanced FCM holds great promise for non-invasive AD diagnosis.

#### 6.1.2. Antibody Microarrays

Antibody microarrays achieve ultrasensitive solid-phase EV quantification by immobilizing capture antibodies on substrates like glass slides or nanowells to isolate EVs via surface antigens, followed by fluorescence detection with labeled probes or signal amplification [142]. Microarrays consume very small sample volumes and can monitor hundreds of targets in parallel. Martel et al. introduced EVPio, a high-throughput antibody microarray method for simultaneous analysis of both inner and outer proteins of EVs [143]. This approach utilizes optimized fixation and heat-induced epitope retrieval, coupled with oligonucleotide-barcoded antibodies and branched DNA amplification, enabling sensitive and multiplexed detection. EVPio can capture EV subpopulations based on surface markers and quantify co-expression of multiple proteins, providing insights into EV heterogeneity and potential applications in disease diagnostics. Additionally, immuno-digital invasive cleavage assay (idICA) combines immunocapture of EVs with single-molecule fluorescence readout in a microarray of nanowells. Yuyama et al. applied idICA to count Aβ-bound EVs: EVs bearing ganglioside GM1 were pulled down on magnetic beads and tagged with DNA-conjugated antibodies; upon specific binding, a cleavage reaction generated fluorescent signals on individual beads, which were counted digitally [144]. This enabled femtogram-level detection of EV-associated Aβ and showed that blood Aβ-positive EVs increased with age in AD model mice, mirroring brain amyloid accumulation. These innovations in antibody microarray detection enable ultrasensitive, cell-type-specific profiling of CNS-derived EVs for AD pathology, thereby advancing early diagnostic capabilities.

Antibody microarrays excel at multiplexed phenotyping of EVs with minimal sample input [142]. They allow parallel detection of dozens of markers, yielding high throughput and sensitivity [145]. SP-IRIS arrays even achieve single-vesicle sensitivity [146]. These microarrays provide semi-quantitative, multiplexed data, which is particularly advantageous for studying EV heterogeneity. However, this approach usually requires purified or enriched EV samples, and it relies on costly instrumentation (e.g., fluorescence scanners and interferometric imaging systems). Clinically, antibody microarrays have translational promise in biomarker discovery and personalized profiling. Commercial kits and instruments are emerging. With further standardization and validation, antibody microarrays could enable multiplex EV biomarker panels for early AD diagnosis and monitoring.

#### 6.1.3. Nucleic Acid Aptamer Sensors

Nucleic acid aptamer sensors use synthetic single-stranded DNA or RNA oligonucleotides to specifically recognize EV surface proteins or internal biomarkers [147,148]. Aptamers can be immobilized on a sensor surface (e.g., electrodes, nanoparticles, and microfluidic chips) to capture EVs, or they can act as signaling probes that bind EV components. Detection can then be performed via fluorescence, electrochemical methods, Raman spectroscopy, or other modes [148]. Similar to antibodies, aptamers often exhibit high binding affinity, but they are less expensive to synthesize, more stable under various conditions, and easier to chemically modify. Zhou et al. reported an enzyme-free entropy-driven strand displacement reaction (ESDR) assay that detects Aβ42 oligomers in exosomes by fluorescently labeled DNA probes—the target Aβ oligomers trigger a cyclic DNA hybridization, yielding a strong signal with a 20 pM detection limit [149]. Building on this, a dumbbell aptamer sensor combined two biomarkers (Aβ42 and miR-193b) for earlier AD detection; by using dual aptamers and graphene oxide (GO) quenching, it achieved limits of detection (LODs) of ~53–77 pM and distinguished a “one increase (Aβ42) and one decrease (miR-193b)” signature in neurogenic exosomes from early AD models [150].

Aptamer sensors generally exhibit high affinity and specificity for their targets [151]. They can be selected to bind a wide variety of biomolecules (e.g., proteins, nucleic acids, and lipids), and their chemical synthesis ensures controllable and reproducible production [148]. Compared to antibodies, aptamers are more resistant to heat and chemical degradation and can be regenerated for repeated use. However, aptamer sensors often require chemical modifications to resist degradation in complex samples such as serum. Clinically, aptamer sensors are well-suited for development into rapid, portable diagnostic platforms [152]. In the future, with the advancement of novel signal amplification strategies and the expansion of aptamer libraries, aptamer-based sensors are expected to enable simple, low-cost, and highly sensitive detection of CNS-derived EVs, particularly well-suited for point-of-care applications and multiplexed parallel detection [147].

### 6.2. SPR-Based Detection

SPR-based detection exploits the resonance oscillation of electrons at a metal surface to transduce biomolecular binding events into an optical signal [153]. In EV research, SPR biosensors allow label-free, real-time quantification of vesicles or their molecular contents [154]. A key advantage is that SPR can detect very low concentrations by signal amplification strategies. For example, Song et al. developed a label-free SPR biosensor using a DNA-assembled plasmonic nano-architecture to capture and detect blood EV miRNAs associated with AD. This sensor featured two nanoscale gaps that enhanced the local refractive index sensitivity 1.66-fold over conventional Au nanorods, enabling attomolar detection of target miRNAs. In tests on clinical serum, it could distinguish AD patients from healthy controls via EV miR-125b and miR-361 levels, achieving a sensitivity of 91.67%, selectivity of 95.00%, and accuracy of 99.52% [155]. Pushing the sensitivity further, Song et al. introduced a curved plasmonic nanostructure with tunable nanogaps. This SPR platform could even stratify patients with MCI from those with AD based on their serum EV miRNA profiles, with an average diagnostic accuracy of 98.22% [156]. Likewise, researchers have developed antibody-functionalized titanium nitride (TiN) SPR biosensors: functionalizing TiN with biotinylated antibodies enables specific capture of exosomes, with plasmonic resonance shifts quantifying sub-µg/mL levels of surface biomarkers in samples [157]. These examples illustrate how SPR can be tailored for various EV targets with extreme sensitivity.

SPR-based detection enables label-free, real-time quantification of EVs and their biomolecular interactions through direct optical transduction [158]. Key advantages include high sensitivity for low-abundance analytes, kinetic monitoring of binding events without secondary labels, and versatility through customizable surface ligands (e.g., antibodies or aptamers). Moreover, advances like nanostructured gold surfaces and coupling with fluorescent or electrochemical readouts further boost sensitivity and specificity [155]. However, SPR technology faces limitations including susceptibility to bulk refractive index interference in complex biofluids, necessitating rigorous calibration and sample preprocessing. Despite these constraints, SPR-based EV assays have demonstrated clinically relevant detection of AD biomarkers in biofluids. Their rapid analysis and minimal sample volumes align with point-of-care requirements [154]. Further development of portable instrumentation, integrated microfluidics for on-chip enrichment, and antifouling surface chemistries could enhance clinical utility for early AD diagnosis.

### 6.3. SERS-Based Detection

SERS is an analytical technique that enhances the Raman scattering of molecules adsorbed on nanostructured metal surfaces [159]. In SERS-based EV detection, the minute vibrational fingerprint signals of EV components are amplified by factors of 105–108 via plasmonic nanostructures. This allows for ultrasensitive, label-free identification of EV molecular profiles [160]. SERS-based methodologies are typically divided into direct and indirect categories. Direct SERS detects the intrinsic Raman spectra of analytes without requiring labeling with Raman reporters, also termed label-free SERS analysis, enabling fingerprint identification of unmodified biomolecules [161]. In one approach, single-exosome Raman fingerprints acquired via graphene-Au pyramid hybrid substrates enabled biological source differentiation after ultracentrifugation-based isolation [162]. Additionally, Stremersch et al. distinguished EVs from disease versus healthy cells by coating vesicles with gold nanoparticles and applying multivariate analysis to single-vesicle SERS spectra [163]. In addition to label-free methods, indirect SERS methods have been developed for higher specificity. In these, molecules are targeted by a recognition element, and a Raman reporter tag is brought in proximity [164]. For instance, Zhang et al. created a SERS platform for multiplexed detection of core AD biomarkers by conjugating peptide aptamers for Aβ oligomers and tau to gold nanoparticles. When these aptamer-AuNP conjugates bound their targets in a sample, they were captured on a SERS-active surface, producing distinct Raman signals for each biomarker [165]. Further exploration of indirect SERS approaches for the detection of CNS-derived EVs in AD is required.

SERS offers significant advantages for EV analysis in clinical settings. Its ability to provide unique molecular fingerprints enables the label-free, multiplexed detection of multiple EV components simultaneously, as each molecule generates distinct Raman peaks [166]. This information-rich spectral output holds promise for discovering novel EV biomarkers. Furthermore, SERS achieves exceptional sensitivity; with optimized substrates, single-EV or even single-molecule detection is feasible. However, key challenges hinder clinical translation: SERS signals suffer from reproducibility issues due to variations in nanostructure fabrication and sample placement, complicating quantification [167]. Interpreting complex EV spectra also demands advanced chemometric tools to deconvolute overlapping signals. Despite these hurdles, the combination of SERS with machine learning for spectral pattern recognition could yield powerful clinical tools [168].

### 6.4. Colorimetric-Based Detection

Colorimetric-based detection for EVs relies on visible color changes triggered by specific EV-target interactions [169]. Common mechanisms include enzyme-substrate chromogenic reactions, plasmonic shifts from nanoparticle aggregation, or DNA/aptamer hybridization affecting dye signals [170]. In EV analysis, immunoassay formats often localize HRP or nanozyme labels to EVs, so that substrate turnover yields a blue/green color proportional to EV amount. Similarly, Au nanoparticle (AuNP) systems exploit aptamer or antibody binding to displace AuNP-stabilizing ligands: target EVs cause AuNP. For instance, Jiang et al. used AuNPs complexed with aptamers so that exosome binding releases aptamers and triggers AuNP aggregation/colorimetric change [171]. Additionally, Zhang et al. developed a nanozyme sandwich assay in plasma: NDEs were magnetically captured and probed by Au@Pt nanozymes bearing Aβ42 aptamers. The sandwich complex catalyzes TMB oxidation to produce a blue signal only in the presence of Aβ42-positive EVs. This sensor achieved high sensitivity over a range of 1 × 10^5^–1 × 10^9^ particles/mL and successfully distinguished AD patient plasma from controls [172]. Similarly, Zhang et al. reported a “SWzyme” assay with anti-CD63 capture on a biochip and Ni@Pt nanozyme detection. They detected plasma exosomes with LOD ~4.2 × 10^4^ particles/mL [173]. A label-free approach by Wu et al. used pollen-derived sporopollenin microcapsules loaded with AuNPs and CD63 aptamer. This assay gave LOD ~10 particles/μL and a wide linear span [174]. These examples span various EV sources, demonstrating the versatility of colorimetric EV assays.

Colorimetric assays are generally low-cost, instrument-free, and easy to read by eye or simple imaging [175]. They integrate well with paper- or microfluidic-based POC devices due to minimal components and rapid visual output. Using nanozymes or enzyme labels yields amplified signals visible in minutes, while AuNP-based methods allow multiplex profiling of EV surface markers by distinct color patterns [172]. However, pure colorimetric methods often have modest sensitivity compared to fluorescence or digital assays. In conclusion, colorimetric assays hold promise for point-of-care applications. Their simplicity and low cost make them appealing for decentralized screening or resource-limited settings [176]. Further optimization of sensitivity and multiplexing, as well as large-scale validation, will be needed to move colorimetric EV diagnostics into clinical use.

### 6.5. Electrochemical-Based Detection

Electrochemical detection translates biomolecular interactions on an electrode surface into measurable electrical signals, enabling highly sensitive detection of CNS-derived EV biomarkers [177,178]. These systems often employ immunocapture or aptamer-based binding of EV surface markers or cargo, followed by electrochemical readouts proportional to target concentration. For example, Li et al. developed an integrated microfluidic EVID-biochip that isolates NDEs with antifouling magnetic beads and then detects the neuron-specific L1CAM protein via an on-chip electrochemical immunosensor. This platform demonstrated high specificity for NDEs, achieving a L1CAM detection sensitivity of 1 pg/mL [179]. Zheng et al. reported a novel bioelectronic platform based on an organic electrochemical transistor (OECT) with a microelectrode array for multi-AD biomarker detection [180]. Another system by Li et al. integrates an OECT with acoustoelectric EV enrichment for rapid, point-of-care testing. Focused acoustic waves concentrate EVs onto the OECT gate functionalized with anti-Aβ/tau antibodies, yielding a LOD of 500 particles/μL and an assay time of 2 min. This device successfully monitored rising Aβ-positive EV levels in an AD mouse model over 18 months, correlating with plaque progression [181].

Electrochemical detection offers several advantages: ultrahigh analytical sensitivity, even in complex biofluids, and relatively fast turnaround by eliminating lengthy amplification or enrichment steps [182]. Moreover, many methods use inexpensive screen-printed electrodes or miniaturized chips, suggesting low per-test cost and compatibility with portable electronics. Electrochemical methods face challenges on the path to clinical translation. Currently, the throughput is moderate. Many prototypes process a single sample in 1–2 h or in small batches, although newer designs greatly reduce assay time to minutes per sample. Integrating microfluidics for automated sample handling can improve throughput. Another consideration is validation and reliability in clinical settings: most systems have been tested on limited cohorts. Larger multi-center studies are needed to confirm robustness across populations. Encouragingly, several electrochemical EV assays have already demonstrated clinically relevant performance, highlighting their translational potential [183,184,185]. With continued refinement, electrochemical detection could enable cost-effective, rapid EV-based diagnostics for AD and other neurodegenerative conditions at the point of care.

### 6.6. Nanomechanical-Based Detection

Nanomechanical detection exploits the physical mass and mechanical effects of EVs binding to a sensor [186]. EVs are captured on a functionalized surface. Each EV binding adds tiny mass or surface stress, leading to a measurable mechanical signal. By monitoring these changes, one can quantify the bound EVs [187]. Cafolla et al. developed a method using vibrating microcantilevers to directly quantify EVs in unprocessed saliva [188]. By operating the cantilever at small oscillation amplitudes in the liquid, they achieved attogram mass resolution even in viscous biofluids. Remarkably, they could detect EV concentrations down to 0.1 µg/mL of saliva within 20 min. Another notable advancement is the magneto-nanomechanical sensor developed by Mei et al. Exosomes were labeled with magnetic nanoparticles, and an external magnetic field was applied to generate an additional force on each bound exosome [189]. This magnetically-enhanced cantilever achieved a five-orders-of-magnitude sensitivity gain compared to standard surface-stress sensors. Acoustic resonators like Quartz Crystal Microbalance with Dissipation (QCM-D) have also been applied to EVs. For example, Suthar et al. used a QCM-D immunosensor to detect exosomes by capturing them with an anti-CD63 antibody on the crystal. They observed measurable frequency shifts and could differentiate exosome concentrations in plasma samples label-free [190]. Another study demonstrated a dual-mode QCM-electrochemical sensor that simultaneously tracked the QCM frequency shift and an impedance change when exosomes bound, improving reliability in complex fluids [191]. Additionally, another approach involved the use of AuNP-labeled antibodies on a cantilever, amplifying the mass change [192]. This allowed detection of EV markers with improved sensitivity.

Nanomechanical detection offers the advantage of being label-free and direct, requiring no enzymatic amplification or fluorescent labeling [186,193]. This reduces assay complexity and potential interference. These sensors measure an inherent property, so they can quantify EVs in absolute terms. However, the throughput has been a limitation: many cantilever setups measure one sample at a time [193]. In summary, nanomechanical detection is rapidly maturing, with sensitivities now approaching or surpassing those of optical and magnetic methods. With continued innovation in sensor design, nanomechanical assays are on a tangible path toward clinical diagnostic tools.

## 7. Discussion

### 7.1. Clinical Utility and Applications

CNS-derived EV biomarkers act as minimally invasive “liquid biopsies” that protect their internal cargo from degradation in biofluids and thus provide a direct window into AD-related brain pathology via simple blood, saliva, or urine assays. If reliable EV assays can be developed, clinicians could use them to detect AD pathology at very early, even pre-symptomatic stages. Routine screening of at-risk individuals (e.g., older patients with mild cognitive complaints or a family history of AD) could become feasible, enabling much earlier diagnosis and timely treatment. Beyond early detection, EV biomarkers could stratify patients and guide personalized interventions aligned with each patient’s specific disease mechanisms. EV-based tests would also permit minimally invasive monitoring of disease progression and therapy response [194]. Serial EV assays could track changes in biomarker levels over time, giving feedback on whether treatments are effectively engaging their targets or slowing disease progression, much like glucose monitors guide diabetes management. For example, an integrated EV sensor has even been shown to track amyloid-bearing EV levels over 18 months in a model, correlating with plaque buildup in the brain [181].

Moreover, EV assays have broad scalability and cost advantages: they could be implemented in primary-care settings to screen patients with mild cognitive symptoms, where a positive EV result would prompt confirmatory PET or CSF testing or treatment, whereas a negative result could help avoid unnecessary procedures. In contrast to current standards—CSF Aβ/tau assays that require lumbar puncture and amyloid PET/MRI imaging that is expensive—EV biomarker tests can be performed in most clinical labs or even at the point of care without specialized equipment, enabling faster rule-in/rule-out decisions at much lower cost. From a patient’s perspective, EV testing is far more acceptable, as it requires only a routine blood, saliva, or urine sample with minimal discomfort. This stands in stark contrast to invasive lumbar punctures or lengthy PET scans with radiation that many patients avoid. Because EV tests are easily repeatable, patients could undergo annual EV monitoring to gauge disease status or treatment response—something virtually impossible with CSF sampling. Routine biofluid collection imposes virtually no barrier to serial testing, and sample accessibility is vastly easier than obtaining CSF. Such accessibility would greatly broaden the reach of early AD detection and prevention strategies. Overall, the successful translation of CNS-derived EV biomarkers into the clinic would represent a leap forward for AD—enabling risk assessment, early intervention, and therapy monitoring tailored to each patient’s disease trajectory [195].

### 7.2. Current Limitations and Challenges

Despite the promise of CNS-derived EVs as AD biomarkers, several formidable hurdles must be overcome before they can be implemented in clinical practice. First, standardization and reproducibility are major concerns: different studies employ diverse EV isolation and analysis protocols, and the lack of standardized methods leads to poor comparability of results across platforms. Second, specificity is a concern: many “brain-specific” EV markers are not exclusively from the CNS, and AD overlaps with other dementias and comorbidities. In other words, EV signatures may not uniquely pinpoint AD pathology and can be confounded by mixed diseases. In addition, CNS-derived EVs are present at extremely low levels in biofluids, posing substantial technical challenges for their specific detection. As a result, enrichment methods may capture unwanted vesicle populations, while any carryover of plasma proteins or lipoproteins can confound downstream biomarker measurements. Third, current EV isolation and detection techniques often lack practicality for routine clinical use. Many “gold-standard” methods are labor-intensive and not easily scalable [91]. For example, ultracentrifugation is time-consuming, requires specialized equipment, and processes only a few samples at a time. Such constraints make it unsuitable for high-throughput screening. Even other isolation approaches, such as SEC and precipitation, face trade-offs between yield and purity that can undermine diagnostic reliability.

These hurdles collectively illustrate the gap between exploratory research findings and a robust, clinic-ready EV diagnostic test. In sum, before EV tests can reach the clinic, field-wide rigor is needed to improve reproducibility, specificity, and analytical robustness.

### 7.3. Emerging Directions and Prospects

Advances in data science are expected to drive the EV biomarker field forward. AI-based methods, especially machine learning, for data integration, are beginning to reveal richer EV signatures. These approaches underscore the power of multi-omics integration: by combining EV proteomes with RNA omics and other omic layers, investigators can identify novel biomarker panels beyond classical amyloid/tau readouts. Equally important are coordinated efforts on standardization and validation, since adherence to common protocols and rigorous quality controls is critical to ensure reproducibility across laboratories. To that end, establishing harmonized guidelines for sample collection, EV isolation, and biomarker analysis is a top priority, including the use of standardized reagents, reference materials, and defined quality-control procedures to ensure consistent results across studies. Meanwhile, large-scale multi-center studies are needed to rigorously evaluate CNS-derived EV biomarkers in diverse patient cohorts and establish normative ranges and clinically relevant cut-off values. Without such consensus efforts and validation studies, promising EV biomarkers will remain at the preclinical stage.

Technological innovation must go hand-in-hand with these efforts. Given that no single EV marker exhibits complete cell specificity, researchers have begun exploring novel approaches to enhance source specificity and reduce contamination [196]. Notably, recent advancements in single-EV analysis now enable high-throughput phenotyping of individual ~100 nm vesicles to infer their cell of origin with high specificity [197]. Applying similar single-vesicle phenotyping approaches in AD could help distinguish CNS-derived EVs from the sea of vesicles in biofluids, effectively improving signal-to-noise. Indeed, using combinations of markers and sensitive detection methods, imaging flow cytometry has demonstrated over 90% specificity in identifying true EV events in plasma [198]. At the same time, microfluidic lab-on-a-chip systems have emerged as a promising solution to practical challenges in EV analysis [199]. These microfluidic devices can work with small sample volumes and low EV concentrations, integrating EV isolation, enrichment, and detection on a single platform [200]. By combining steps, such integrated devices improve throughput and consistency and can be automated. Other emerging strategies like aptamer-based sensors and nanomechanical detectors are also being investigated for integration into microfluidic platforms. By miniaturizing and automating these processes, operator-dependent variability is minimized and clinical scalability is improved. Ultimately, these technological advances aim to produce EV detection platforms that are fast, reliable, and easy to use in clinical settings.

With continued research and concerted effort on standardization, validation, and innovation, CNS-derived EVs may soon fulfill their promise as a reliable clinical tool for AD, becoming a cornerstone of next-generation precision diagnostics and personalized patient care.

## Figures and Tables

**Table 1:** Potential CNS-derived EV biomarkers in the detection of AD.

Biomarkers	Source	EV Subpopulation	Surface Marker	Findings in AD vs. Controls	**References**
Amyloid-related biomarkers					
Aβ42, Aβ40, Aβ1-42	Plasma	NDEs	L1CAM, GAP43, NLGN3, NCAM	Aβ42, Aβ1-42: MCI, AD (↑) Aβ42/40: AD (↑)	[56,57,58,59,60,61,62]
Tau pathology biomarkers					
P-T181-tau, P-S396-tau, P-T231-tau, T-tau	Plasma	NDEs	L1CAM, NCAM, GAP43, NLGN3, amphiphysin 1	P-T181-tau, P-S396-tau, T-tau: MCI, AD (↑) P-T231-tau: AD (↑)	[56,57,59,60,61,62,63,64]
Neurodegeneration/neuronal injury biomarkers					
synaptophysin, synaptopodin, synaptotagmin-1, synaptotagmin-2, SNAP-25, syntaxin-1, NRGN, GAP43, PSD95, GluR2, AMPA4, NPTX2, NLGN1, NRXN2α, REST, proBDNF	Plasma Serum	NDEs	L1CAM, NCAM, GAP43, NLGN3	synaptotagmin-1, SNAP-25, NRGN, GAP43, REST: MCI, AD (↓) synaptophysin, synaptopodin, synaptotagmin-2, syntaxin-1, PSD95, GluR2, AMPA4, NPTX2, NLGN1, NRXN2α, proBDNF: AD (↓)	[56,60,64,65,66,67,68]
Inflammatory/immune mechanism-related biomarkers					
C1q, C4b, C3b, C3d, factor B, factor D, fragment Bb, C5b-C9 TCC, IL-6, TNF-α, IL-1β, CD59, CD46, DAF, CR1, MCP-1, HGF, FGF-2, FGF-13, IGF-1	Plasma	ADEs	GLAST	C1q, C4b, C3b, C3d, factor B, factor D, fragment Bb, C5b-C9 TCC, IL-6, TNF-α, IL-1β: AD (↑) CD59, CD46, DAF, CR1, HGF, FGF-2, FGF-13, IGF-1: AD (↓) MCP-1: SCD (↓)	[69,70,71]
C7, HGF, FGF-13, IGF-1, MMP-9	Plasma Serum	NDEs	L1CAM	C7, MMP-9: AD (↑) HGF, FGF-13, IGF-1: AD (↓)	[59,71,72]
Vascular brain injury-related biomarkers					
Hemoglobin, Hemoglobin subunit α, β, and δ	Plasma	NDEs	L1CAM	Hemoglobin, Hemoglobin subunit α, β, and δ: AD (↑)	[73]
Other biomarkers—proteins					
ZYX, pY-IRS-1, p-S312-IRS-1, p-panY-IRS-1, SOD1, mitochondrial electron transport chain complexes I, III, IV, ATP synthase, cathepsin D, LAMP-1, ubiquitin, HSP70	Plasma Serum	NDEs	L1CAM	pY-IRS-1, p-S312-IRS-1, cathepsin D, LAMP-1, ubiquitin: AD (↑) ZYX, p-panY-IRS-1, SOD1, mitochondrial electron transport chain complexes I, III, IV, ATP synthase, HSP70: AD (↓)	[63,72,74,75,76]
Other biomarkers—proteins					
BACE1, sAPPβ, GDNF	Plasma	ADEs	GLAST	BACE1, sAPPβ: AD (↑) GDNF: AD (↓)	[77]
Other biomarkers—microRNAs					
miR-384, miR-29c-3p, miR-let-7e-5p, miR-122, miR-3591, miR-9-5p, miR-106b-5p, miR-125b-5p, miR-132-5p, miR-29a-5p, miR-210-3p, miR-212-3p, miR-132-3, miR-23a-3p, miR-223-3p, miR-190-5p, miR-100-3p	Plasma	NDEs	L1CAM, NCAM, amphiphysin 1	miR-384, miR-29c-3p, miR-let-7e-5p, miR-9-5p, miR-106b-5p, miR-125b-5p, miR-132-5p, miR-23a-3p, miR-223-3p, miR-190-5p: AD (↑) miR-122, miR-3591, miR-29a-5p, miR-212-3p, miR-132-3, miR-100-3p: AD (↓) miR-210-3p: MCI (↑)	[61,62,78,79,80,81,82]
miR-29a-5p, miR-107, miR-125b-5p, miR-132-5p, miR-210-3p	Plasma	ADEs	GLAST	miR-29a-5p, miR-107, miR-125b-5p, miR-132-5p: AD (↑) miR-210-3p: MCI (↑)	[80]
miR-29a-5p, miR-125b-5p, miR-132-5p, miR-210-3p, miR-106b-5p	Plasma	MDEs	TMEM119	miR-29a-5p: AD (↓) miR-125b-5p: MCI (↓) miR-132-5p, miR-106b-5p: AD (↑) miR-210-3p: MCI-AD (↑)	[80]

Abbreviations: Aβ, amyloid beta; P-T181-tau, phosphorylated tau at threonine 181; P-S396-tau, phosphorylated tau at serine 396; P-T231-tau, phosphorylated tau at threonine 231; T-tau, total tau; SNAP-25, synaptosomal-associated protein 25; NRGN, neurogranin; PSD95, postsynaptic density protein 95; GluR2, glutamate receptor 2; AMPA4, AMPA receptor subunit 4; NPTX2, neuronal pentraxin 2; NLGN1, neuroligin 1; NRXN2α, neurexin 2 alpha; REST, RE-1 silencing transcription factor; proBDNF, precursor brain-derived neurotrophic factor; C1q, complement component 1q; C4b, complement component 4b; C3b, complement component 3b; C3d, complement component 3d; factor B, complement factor B; factor D, complement factor D; fragment Bb, complement factor B fragment Bb; C5b-C9 TCC, terminal complement complex, comprising complement components C5b-C9; IL, interleukin; TNF-α, tumor necrosis factor-α; DAF, decay-accelerating factor; CR1, complement receptor 1; MCP-1, monocyte chemoattractant protein-1; HGF, hepatocyte growth factor; FGF, fibroblast growth factor; IGF-1, insulin-like growth factor 1; C7, complement component 7; MMP-9, matrix metalloproteinase 9; ZYX, zyxin; pY-IRS-1, tyrosine-phosphorylated insulin receptor substrate 1; p-S312-IRS-1, insulin receptor substrate 1 phosphorylated at serine 312; p-panY-IRS-1, pan-tyrosine-phosphorylated insulin receptor substrate 1; SOD1, superoxide dismutase 1; LAMP-1, lysosome-associated membrane protein 1; HSP70, heat shock protein 70; BACE1, β-site amyloid precursor protein-cleaving enzyme 1; sAPPβ, soluble amyloid precursor protein β; GDNF, glial cell line-derived neurotrophic factor; miRNA, microRNA; NDEs, neuron-derived extracellular vesicles; ADEs, astrocyte-derived extracellular vesicles; MDEs, microglia-derived extracellular vesicles; L1CAM, L1 cell adhesion molecule; GAP43, growth-associated protein 43; NLGN3, neuroligin 3; NCAM, neural cell adhesion molecule; GLAST, glutamate-aspartate transporter; TMEM119, transmembrane protein 119; MCI, mild cognitive impairment; AD, Alzheimer’s disease; SCD, subjective cognitive decline. Note: “↑” indicates an increase in biomarker level; “↓” indicates a decrease in biomarker level.

**Table 2:** Comparison of CNS-derived EV isolation methods.

Isolation Techniques	Principle	Yield	Purity	Scalability	Throughput	Time	Advantages	**Disadvantages**	**References**
Ultracentrifugation-Based Separation	Separates EVs based on their buoyant density using ultracentrifugation or density gradients	Low to moderate	High	Low	Low	Long	Well-established; suitable for bulk EV isolation; high yield	Time-consuming; labor-intensive; large sample volume	[89,90,91,92]
Size-Based Separation	Filters or retains EVs based on size using ultrafiltration membranes or SEC columns	Moderate to high	Moderate to high	High	Moderate	Short	Preserves EV integrity; mild conditions	Risk of filter clogging; co-isolation of similar-sized contaminants	[93,94,95]
Precipitation Methods	Uses polymers to precipitate EVs by reducing their solubility	High	Low	High	High	Moderate	Simple; low-cost; compatible with clinical workflows	Low specificity; potential contamination by non-EV proteins or particles	[96,97,98]
Charge-/Dielectric-Based Separation	Uses charge or dielectric differences to separate EVs through electrophoresis or dielectrophoresis	Moderate	High	Moderate to high	Low	Moderate	High purity; potential for fast sorting	Low throughput; less established for clinical use	[99,100,101]
Immunoaffinity-Based Separation	Employs antibodies specific to capture target EVs via magnetic beads or affinity columns	Low to moderate	High	Low to moderate	Low	Moderate	High specificity; enriches cell-type or disease-specific EVs	High-cost; limited to known markers	[102,103,104]
Acoustic Separation	Applies acoustic forces to separate EVs based on size and compressibility	Moderate to high	High	Moderate	Low to moderate	Short	Contact-free; gentle processing; compatible with continuous flow	Requires precise instrumentation; low throughput	[105,106,107]
